# Systemic and local effect of the *Drosophila headcase* gene and its role in stress protection of Adult Progenitor Cells

**DOI:** 10.1371/journal.pgen.1009362

**Published:** 2021-02-08

**Authors:** Panagiotis Giannios, Jordi Casanova

**Affiliations:** 1 Institut de Biologia Molecular de Barcelona (CSIC), Barcelona, Catalonia, Spain; 2 Institut de Recerca Biomèdica de Barcelona, (IRB Barcelona), The Barcelona Institute of Science and Technology (BIST), Barcelona, Catalonia, Spain; New York University, UNITED STATES

## Abstract

During the development of a holometabolous insect such as *Drosophila*, specific group of cells in the larva survive during metamorphosis, unlike the other larval cells, and finally give rise to the differentiated adult structures. These cells, also known as Adult Progenitor Cells (APCs), maintain their multipotent capacity, differentially respond to hormonal and nutritional signals, survive the intrinsic and environmental stress and respond to the final differentiation cues. However, not much is known about the specific molecular mechanisms that account for their unique characteristics. Here we show that a specific *Drosophila* APC gene, *headcase* (*hdc*), has a dual role in the normal development of these cells. It acts at a systemic level by controlling the hormone ecdysone in the prothoracic gland and at the same time it acts locally as a tissue growth suppressor in the APC clusters, where it modulates the activity of the TOR pathway and promotes their survival by contributing in the regulation of the Unfolded Protein Response. We also show that *hdc* provides protection against stress in the APCs and that its ectopic expression in cells that do not usually express *hdc* can confer these cells with an additional stress protection. Hdc is the founding member of a group of homolog proteins identified from *C*. *elegans* to humans, where has been found associated with cancer progression. The finding that the *Drosophila hdc* is specifically expressed in progenitor cells and that it provides protection against stress opens up a new hypothesis to be explored regarding the role of the human Heca and its contribution to carcinogenesis.

## Author summary

In all organisms there are sets of “reserve” cells that have the capacity to self-renew and eventually differentiate to repair or provide new structures. Here we report that the *Drosophila headcase* gene provides such kinds of cells with a stress protective mechanism. Interestingly, *headcase* is also involved in providing such “reserve” cells with the signal required for initiating their differentiation into new structures. Thus, *headcase* establishes a fine equilibrium between differentiation and stress protection of these “reserve” cells. Of note *headcase* is the founding member of a group of homolog proteins identified in many animals. In humans, the *headcase* homolog is associated with different kinds of cancers but its function and role remains unknown. The finding that the *Drosophila headcase* confers stress protection opens up a new way to be explored regarding the role of the human Headcase as it could also act as a stress protector and its drop-off may induce stress conditions favouring the initiation and progression of tumorigenesis.

## Introduction

Multipotency has been widely studied in biomedical research, particularly due to its role in tissue maintenance and repair. However, there is a lack of understanding regarding how multipotent cells lead the physiological development of an organism and, most importantly, about the molecular characteristics that allow them to overcome challenges, withstand stress conditions, avoid death and eventually contribute to tissue morphogenesis and sustenance. Here we address some of these issues by exploiting a naturally occurring population of multipotent cells, namely Adult Progenitor Cells (APCs) in *Drosophila melanogaster (Drosophila)*[1,2].

In *Drosophila*, most larval cells are polyploid and they die at the transition between larval and adult stages. In contrast, APCs survive throughout the developmental process and give rise to adult structures [3,4]. These cells are specified during embryonic development, and they undergo several mitotic divisions during larval stages, remain diploid, and finally proceed into their terminal differentiation during pupal metamorphic stages. Both APCs and larval tissue cells are exposed to the same nutritional and hormonal cues, thereby suggesting that unique molecular components act within the APCs to differentially regulate the effect of external and intrinsic stimuli in their unique setting.

The *headcase (hdc)* gene was originally identified by its specific expression in *Drosophila* APCs, and *hdc* mutants failed to emerge as adults, dying in metamorphosis. However, clusters of APCs with seemingly normal size and shape were still found to be present in *hdc* mutant pupae suggesting that *hdc* was not required for the proliferation of APCs, but rather for their adult differentiation [5]. While a very recent report has also identified that *hdc* participates in the response to nutrient restriction [6], we still lack a characterization of the role of such a specific component of APCs in the normal conditions of development.

Here we show a dual role for *hdc* in APCs. On the one hand, we show that *hdc* has a systemic role by acting on the prothoracic gland and regulating the appropriate levels of the hormone ecdysone, which controls the main transitions between developmental stages, including metamorphosis from the last larval instar to the adult organism. On the other hand, we show that there is a specific role of *hdc* in the APCs regulating their survival, growth and proliferation. In this regard, we show that *hdc* acts on APCs by modulating the effect of the dTOR pathway, while in the absence of this gene APCs die through apoptosis, possibly as a result of chronic activation of the Unfolded Protein Response (UPR). In particular, *hdc* confers progenitor cells specific control of the UPR, a mechanism known to protect cells against stress conditions.

## Results

### HDC is required in the prothoracic gland to control the systemic effect of ecdysone

**Fig 1:**
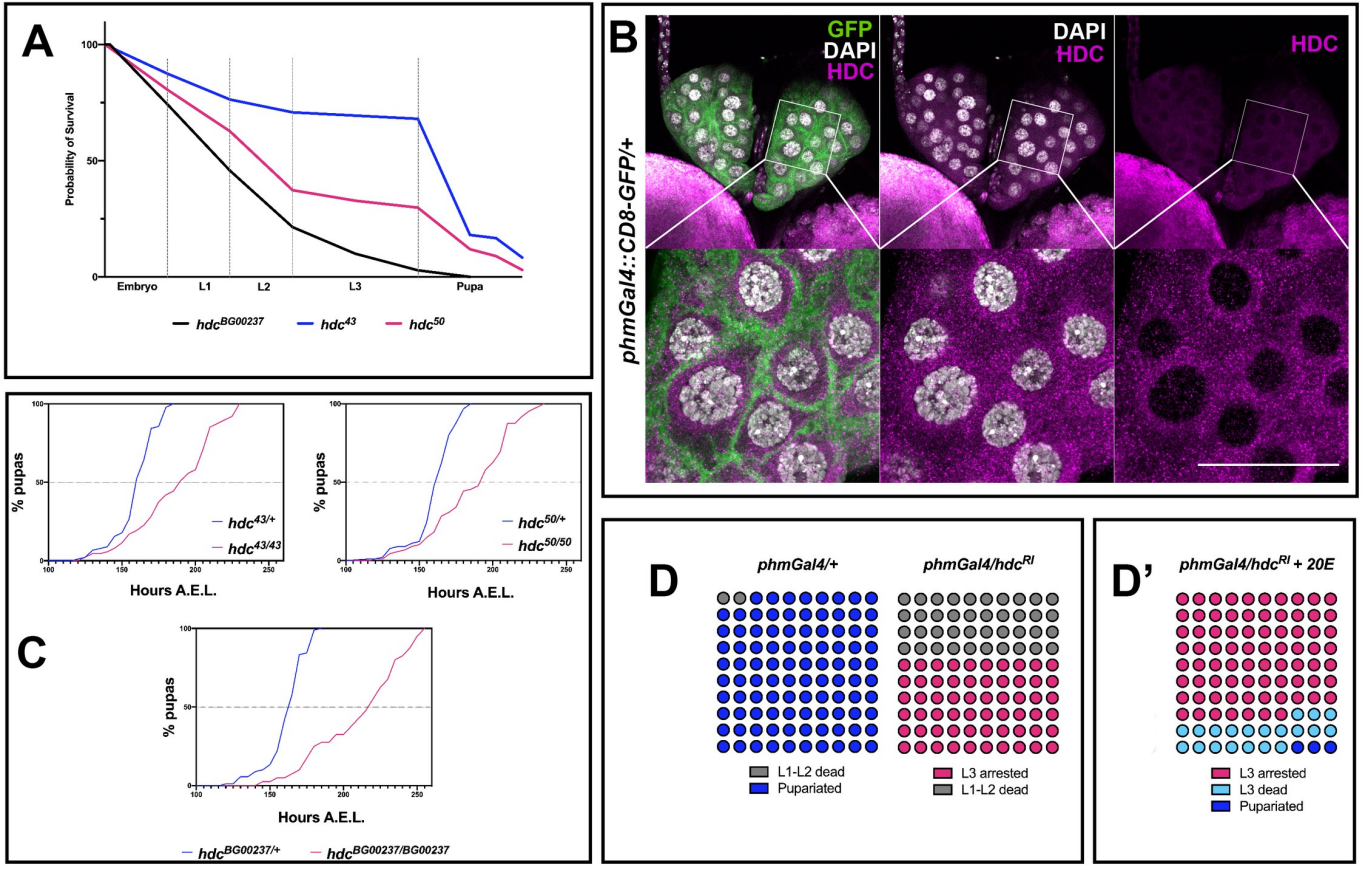
*headcase* is expressed in the prothoracic gland and controls molting in *Drosophila*. **(A)** Survival curves of the three *hdc* mutant strains used in this study. 67%, 35% and 3% of *hdc*^*43*^, *hdc*^*50*^ and *hdc*^*BG00237*^ respectively, reach the pupal stage. For the construction of the curves, 30 individuals were used per strain in 3 replicates, yielding a total of 90 individuals for each of the three *hdc* alleles. **(B)** Hdc protein is detected in the cytoplasmic region in cells of the PG of larval L3 stage at 120 h AEL. **(C)** *hdc* mutants show delayed pupariation. Percentages of pupariated *hdc*^*43/+*^, *hdc*^*50/+*^ and *hdc*^*BG00237/+*^ controls and *hdc*^*43/43*^, *hdc*^*50/50*^ and *hdc*^*BG00237/BG00237*^ mutants are shown at indicated stages. Fifty individuals were tested in each group. **(D)** Knocking down *hdc* in the PG results in larval arrest at the L3 stage and lethality during the L1 and L2 stages (Fisher’s exact test *p<0*.*0001*). **(D’)** 20E feeding rescues developmental arrest for 23% of the surviving L3 instars (Fisher’s exact test *p<0*.*0001*), which die at the transition to pupariation or during early pupal stage. Circles indicate individuals used for each experimental group (n = 100 / experimental condition).

The three *hdc* mutants [*hdc*^*43*^ and *hdc*^*50*^ (partial deletions of the coding region resulting from imprecise P-element excision, described in [5]), *hdc*^*BG00237*^ (P-element insertion resulting in truncated protein, described in [7])] that we analyzed are lethal either as homozygous or over a deficiency (*Df (3R)BSC503*, *Df(3R)ED6332*). Although all *hdc* mutants died as pupae or pharate adults, we observed that many of the mutant individuals failed to reach the pupation and arrested their development at embryonic or larval stages. These observations suggest that *hdc* function is required throughout the development of *Drosophila* (Fig 1A). Interestingly, the death pattern of *hdc* mutants is highly reminiscent of phenotypes associated with loss of 20-hydroxyecdysone (20E)[8], which is produced and released by the prothoracic gland, where *hdc* protein was also detected (Fig 1B). Given that the pulses of 20E in *Drosophila* regulate the major developmental transitions that lead to the final differentiation events before adult eclosion, the death patterns we report are coherent with previous studies that suggest the involvement of *hdc* in developmental events under hormonal control [5,7,9]. Along these lines, we also detected a delay in the *hdc* mutant larvae that eventually reached the pupal stage, another feature associated with lower amounts of ecdysone (Fig 1C).

To study a putative role of *hdc* in ecdysone control mechanisms, we specifically knocked down the gene in the cells of the prothoracic gland (PG). To this end, we used the *phantom22* Gal4 driver (*phmGal4*) to achieve expression of a UAS-*hdc*RNAi (VDRC ID: 104322 construct) (from now on referred to as *hdc*^*RI*^). We observed that 40% of the *phmGal4/hdc*^*RI*^ larvae died at L1-L2 stages (from now on L1 and L2) while the remaining 60% reached the L3 stage (L3) and arrested at this stage for up to 10 days after egg laying (AEL), thus failing to reach the pupal stage (Fig 1D). To exclude the possibility of putative off-target effects caused by RNAi and of non-specific tissue effects caused by the GAL4, on the one hand we knocked down *hdc* in the PG using *phmGal4* to drive the expression of a second UAS-*hdc*RNAi (VDRC ID: 45069 from now on referred to as *hdc*^*R2*^), and on the other hand, we drove the expression of either *hdc*^*RI*^ or *hdc*^*R2*^ in the PG by means of another Gal4 construct (*amnGAL4*) [10]. All the cases mentioned reproduced the death phenotype of L1-L2 and the arrest phenotype of L3, although a small percentage of *amnGal4/hdc*^*R2*^ larvae pupated and then died within the first 10 h of pupal formation (S1 Fig). Administration of 20E through the fly food rescued arrest of *phmGal4/hdc*^*RI*^ L3 larvae (*p<0*.*0001*) and led to the formation of hard cuticle pupal structures for 20% of the individuals at L3. These individuals still failed to progress to pupariation and died shortly after, with the exception of 3%, which entered pupariation and died during white pupa stages (Fig 1D’).

**Fig 2:**
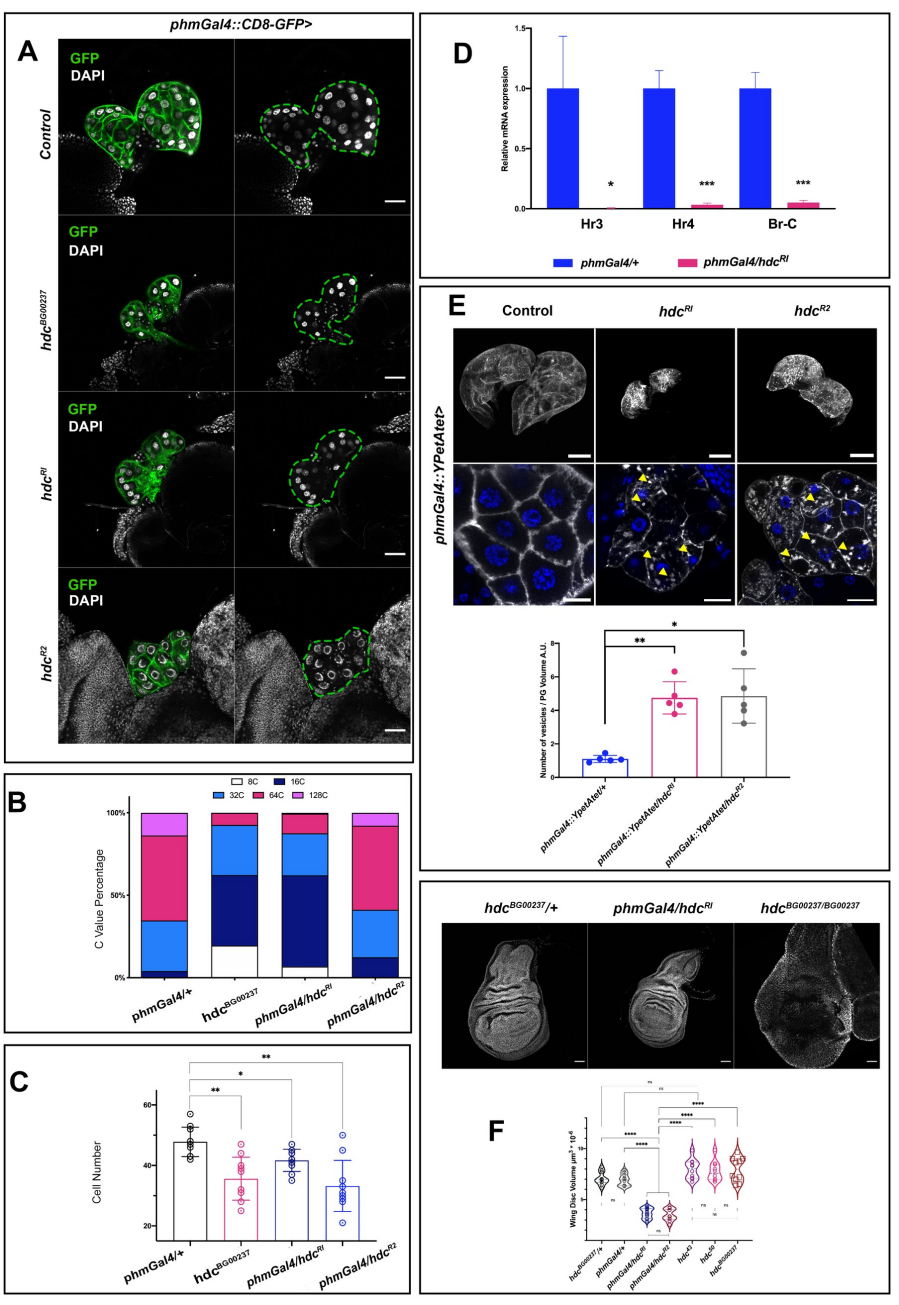
*headcase* affects the physiology of the prothoracic gland and the release of ecdysone. **(A)** Lack of *hdc* in the PG results in smaller glands with fewer cells and a reduced DNA content. Indicative phenotypes of control *phaGal4*::*CD8-GFP/+*, mutant *hdc*^*BG00237*^:: *phaGal4*::*CD8-GFP/ hdc*^*BG00237*^:: *phaGal4*::*CD8-GFP* and *phaGal4*::*CD8-GFP* /*hdc*^*RI*^ and *phaGal4*::*CD8-GFP* /*hdc*^*R2*^ gene knockdowns of L3 larvae at 120 h AEL are shown. Scale bars 40 μm. **(B)** Graph showing the C value of PG cells from each group tested. Depletion of *hdc* induces a reduction in the polyploidy. **(C)** Bar graphs show PG cell count. Depletion of *hdc* results in a lower cell count. For the analysis of variance between groups, Welch’s ANOVA (*p = 0*.*0003*) was applied followed by Dunnett’s T3 multiple group comparison test. (alpha set *at 0*.*05*. * p<0.*05*, **p<0.*005*. n = 10 per group. Error bars represent SD of means). (D) Relative expression levels for the mRNAs of the ecdysone targets *Hr3*, *Hr4* and *Br-C* in total larval extracts appear significantly decreased in *phmGal4/hdc*^*RI*^ larvae compared to control *phmGal4/+ larvae* (alpha set at *0*.*05*. * p<0.*05*, ***p<0.*001*, Unpaired t-test, Welch’s correction) **(E)** PGs of control, *hdc*^*RI*^ and *hdc*^*R2*^ overexpressing YPet-Atet (*phm22>YPet-Atet*) at 120 h AEL. Magnified view in lower panels, shows the aggregation of small vesicle like structures (yellow arrowheads) in the cytoplasm of *hdc-*depleted cells compared to the respective control. Bar charts show comparisons for the ratio of the number of vesicles per PG volume in control and *hdc* knock downs. For the analysis of variance between groups, Welch’s ANOVA (*p = 0*.*0004*) was applied followed by Dunnett’s T3 multiple group comparison test. (alpha set *at 0*.*05*. * p<0.*05*, **p<0.*005*. n = 5 per group. Error bars represent SD of means). Scale bars, upper panels 40 μm, lower panels 20μm. **(F)** Wing discs of control (*hdc*^*BG00237/+*^), *hdc* knockdown in the prothoracic gland (*phmGal4/hdc*^*RI*^) and *hdc* mutant (*hdc* ^*BG00237/ BG00237*^) larvae at 120 h AEL. Violin plots show the distribution of the values for the wing disc volume of controls (*hdc*^*BG00237/+*^ and *phmGal4/+*), *hdc* knockdown in the prothoracic gland (*phmGal4/hdc*^*RI*^; *phmGal4/hdc*^*R2*^), and three different homozygous *hdc* mutants (*hdc*^*43*^, *hdc*^*50*^ and *hdc*^*BG00237*^). The wing disc volume of *phmGal4/hdc*^*RI*^ and *phmGal4/hdc*^*R2*^ larvae is significantly lower (mean = 3.6μm^3^*10^−6^ and 3.5μm^3^*10^−6^) than the control (mean = 6.9 μm^3^*10^−6^ and 7.1 μm^3^*10^−6^ for the *phmGal4/+* and the *hdc*^*BG00237/+*^ respectively) and all mutant groups (mean = 7.9 μm^3^*10^−6^, 7.8 μm^3^*10^−6^ and 7.9 μm^3^*10^−6^, for the *hdc*^*43*^, *hdc*^*50*^ and *hdc*^*BG00237*^ respectively). For the analysis of variance between groups, Welch’s ANOVA (*p<0*.*0001*) was applied, followed by Dunnett’s T3 multiple group comparison test. (**** p<0.*0001*). Interquartile range and mean are indicated by dashed lines. n = 10 per group.

The phenotypes described are indicative of a systemic role of *hdc* through its effect on the PG. To further explore this possibility, we dissected and examined the morphology of PGs from *hdc*^*BG00237*^ L3 larvae at 120h AEL (Fig 2A). The *hdc*-deficient PG cells showed lower cell count and DNA content. The cells of wild-type PGs normally undergo multiple rounds of endocycling—a biological process that is directly linked to the production of ecdysteroids [11]—leading to polyploid nuclei with chromatin values (C Values) of ≥4C. The PGs of *hdc*^*BG00237*^ mutants showed a reduction in nuclear size, a decrease in the C value and a lower total cell count (Fig 2B and 2C). Similar results were observed for the PGs of *phmGal4/hdc*^*RI*^ larvae (Fig 2A and 2C), the majority of their cells remaining at 16C, compared to 64C in the *phmGAL4/+* controls at 120h AEL (Fig 2B), and for the PGs of *phmGal4/hdc*^*R2*^ larvae, where mainly the cell count of the tissue was affected (Fig 2B and 2C).

The general phenotypes observed upon *hdc* depletion in the PG together with the abnormalities in the PG itself, are suggestive of a problem in ecdysone production or secretion. To confirm this hypothesis, we analyzed the expression of ecdysone target genes in control *phmGal4/+* and *phmGal4/hdc*^*RI*^ larvae at 120h AEL. Consistently, expression of *Hr3*, *Hr4* and *Br-C* genes, three direct targets of the hormone that have been used as readouts of the ecdysone levels [12–15] was detected significantly lower, as quantified by qRT-PCR, in *phmGal4/hdc*^*RI*^ larvae compared to the respective controls (Fig 2D).

We also examined whether the effects of *hdc* on the physiology of the ring gland could also extend to the proper secretion of the steroids from the cells of the gland, an additional key mechanism for ecdysone control of developmental transitions [16]. To this end, we knocked down *hdc* in the PGs, together with a construct driving the expression of a fluorescent-protein tagged Atet, which serves as a marker of secretory ecdysone-containing vesicles in the PG [16]. For both *hdc*^*RI*^ and *hdc*^*R2*^, we detected abnormal aggregation of vesicle-like structures along the membrane and the cytoplasm of the PG cells (Fig 2E), indicating an impairment of the hormone secretion mechanism, an additional defect likely to contribute to impaired ecdysone signaling.

Since ecdysone promotes imaginal disc growth, lower levels of this hormone give rise to smaller imaginal discs [17–20]. Accordingly, we observed a significant decrease in the volume of the imaginal discs from *phmGal4/hdc*^*RI*^ larvae at L3 when compared to *phmGAL4/+* controls (Fig 2F); thus, *hdc* activity in the PG non-autonomously promotes the growth of the APC clusters. However, this finding was unexpected given previous reports that suggest either no major effects of *hdc* on imaginal tissue size [5] or even *hdc* acting to restrict tissue growth [6]. Consequently, we decided to also examine the volume of wing discs from *hdc*^*43*^, *hdc*^*50*^ and *hdc*^*BG00237*^ homozygous mutants and compare them to those dissected from *phmGal4/+*, *phmGal4/hdc*^*RI*^ *and phmGal4/hdc*^*R2*^ wandering L3 larvae (Fig 2F). Notably, *hdc* mutant discs sporadically show variable morphological defects, while their mean size was not significantly different compared to the *phmGal4/+* control and was significantly larger than that of *phmGal4/hdc*^*RI*^ *and phmGal4/hdc*^*R2*^ larvae. We interpret these results as an indication of a dual effect of *hdc* on imaginal disc growth control, namely a systemic effect promoting growth and an imaginal disc-specific effect restricting growth.

### HDC is required for the survival of clusters of APCs and the control of adult tissue size

**Fig 3:**
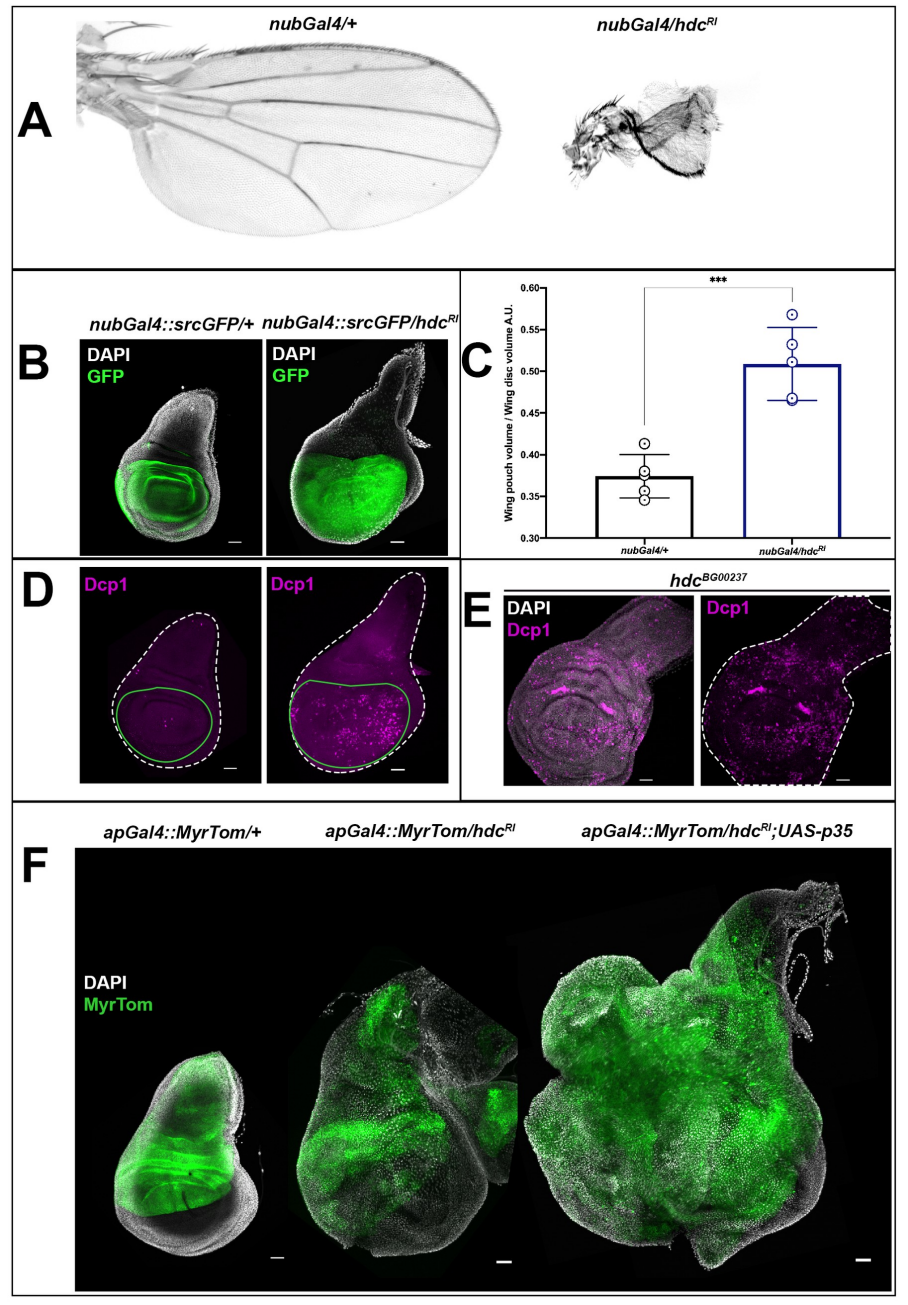
*headcase* is necessary for the survival of imaginal cells and the control of their growth. **(A)** The knockdown of *hdc* in the pouch region of the wing disc results in a significant reduction of the adult wing size and deformation of the wing structure. Adult wings from control (*nubGal4/+*) and *hdc* knockdown (*nubGal4/hdc*^*RI*^) individuals are shown. **(B and C)** The knockdown of *hdc* in the pouch region of the wing disc leads to a significant increase in the size of the wing disc area where *hdc*RNAi is expressed. Wing discs from control (*nubGal4;srcGFP/+*) and *hdc* knockdown (*nubGal4;srcGFP/hdc*^*RI*^) labeled for Src-GFP that marks the pouch region and nuclei (DAPI) of late L3 larvae are shown in **(B)**. In **(C),** quantification of the wing disc pouch volume/wing disc volume ratio in control (*nubGal4/+*, ratio = 0.37) and *hdc* knockdown (*nubGal4/hdc*^*RI*^, ratio = 0.51), reveals the size increase in the area where *hdc* is knocked down. Unpaired t-test, Welch’s correction (*** *p<0*.*001*), n = 5 / group. Error bars represent SD of means. Scale bars, 40 μm. **(D and E)** *hdc* loss results in the induction of apoptosis, as indicated by the Dcp1 marker. Knockdown of *hdc* in the pouch region of the wing disc results in increased Dcp1 staining in the wing disc area where *hdc*RNAi is expressed (wing discs outlined in white dashed line and the wing pouch area where hdc in knocked down in green). Wing discs from control (*nubGal4;srcGFP/+*) and *hdc* knockdown (*nubGal4;srcGFP/hdc*^*RI*^) labeled for Dcp1 (magenta) of late L3 larvae are shown in **(D)**. Extensive Dcp1 staining (magenta) in *hdc* mutant wing discs (*hdc*^*BG00237*^) of late L3 larvae is shown in **(E)**. Nuclei of wing disc cells are labeled by DAPI (white). Scale bars, 40 μm. **(F)** Blocking apoptosis together with *hdc* knockdown results in tumorous overgrowth. Wing discs of control (*apGal4*::*MyrTom/+*), *hdc* knockdown in the apterous expressing region of the wing disc (*apGal4*::*MyrTom/hdc*^*RI*^) and *hdc* knockdown together with the expression of a *p35* construct in the same region (*apGal4*::*MyrTom/hdc*^*RI*^*;UAS-p35*) of late L3 larvae. The *apterous* Gal4 region is labeled by Myristoylated Tomato (pseudo colored green) and cell nuclei are labeled by DAPI stain. Scale bars, 40 μm.

To examine the role of *hdc* in APCs, we specifically knocked down the gene in the pouch region of the wing disc by means of a *nubbin*-Gal4 (*nubGal4*) construct to drive the expression of the *hdc*^*RI*^ construct. The *nubGal4/hdc*^*RI*^ adults showed severe wing malformations, presenting a significant reduction in adult wing size (in 100% of the adults) (Fig 3A). This phenotype was restored after concomitant expression of a full-length mRNA *UAS-hdc* (*hdc*^*UAS*.*cSa*^) construct (S2A Fig). The *hdc*^*R2*^ adults showed milder wing deformations (in 30%), with severe wing malformations appearing in a much lower percentage (4%), suggesting lower gene silencing efficiency (for a detailed characterization of RNAi constructs against *hdc*, see also S2 Fig). Phenotypes of tissue loss in adult *hdc* mutants could not be observed as no escapers survived in homozygosis until the adult stage. Nevertheless, loss of head structures has been observed in pharate adults dissected from their pupal cases [5], while we identified loss of thoracic structures and deformed wing and leg structures from pharate *hdc*^*43*^ and *hdc*^*50*^ mutant escapers.

In contrast to the adult wing phenotypes of *nubGal4/hdc*^*RI*^, we observed an overgrowth in the wing discs from L3 larvae of the same genotype (Fig 3B). In particular, the mean pouch/disc volume ratio, which was 0.37 in control L3 wing discs, increased to 0.51 in the wing discs of *nubGal4/hdc*^*RI*^ L3 larvae at 120h AEL (Fig 3C). We then considered whether the discrepancy between wing disc overgrowth in the larvae and tissue size reduction in the adult, either upon *hdc* RNAi or in *hdc* mutants, might be due to cell loss from the larva to the adult. Indeed, we observed a significant increase in the levels of the effector caspase Dcp1, as assayed with an antibody for this apoptotic marker, both upon *hdc*^*RI*^ expression and in *hdc*^*BG00237*^ imaginal discs (Fig 3D and 3E). This result is similar to that previously reported for *hdc* loss in the stem cells of the adult gut and testis’ hub [21,22]. Thus, our results suggest that *hdc* activity in APC clusters restricts growth and/or proliferation but that the overgrowth elicited upon *hdc* loss is limited because at the same time *hdc* loss also triggers cell death by apoptosis. To corroborate this hypothesis, we induced the expression of the apoptosis inhibitor p35 together with *hdc*^*RI*^ by means of the *ap*Gal4 wing driver. The discs from these larvae, which died at late L3 or early pupal stages, showed aberrant growth similar to that found in tumorigenic conditions (Fig 3F). Thus, the loss of *hdc* in APC clusters promotes both uncontrolled growth and apoptosis.

### Loss of hdc results in the hyperactivation of TOR in APC

**Fig 4:**
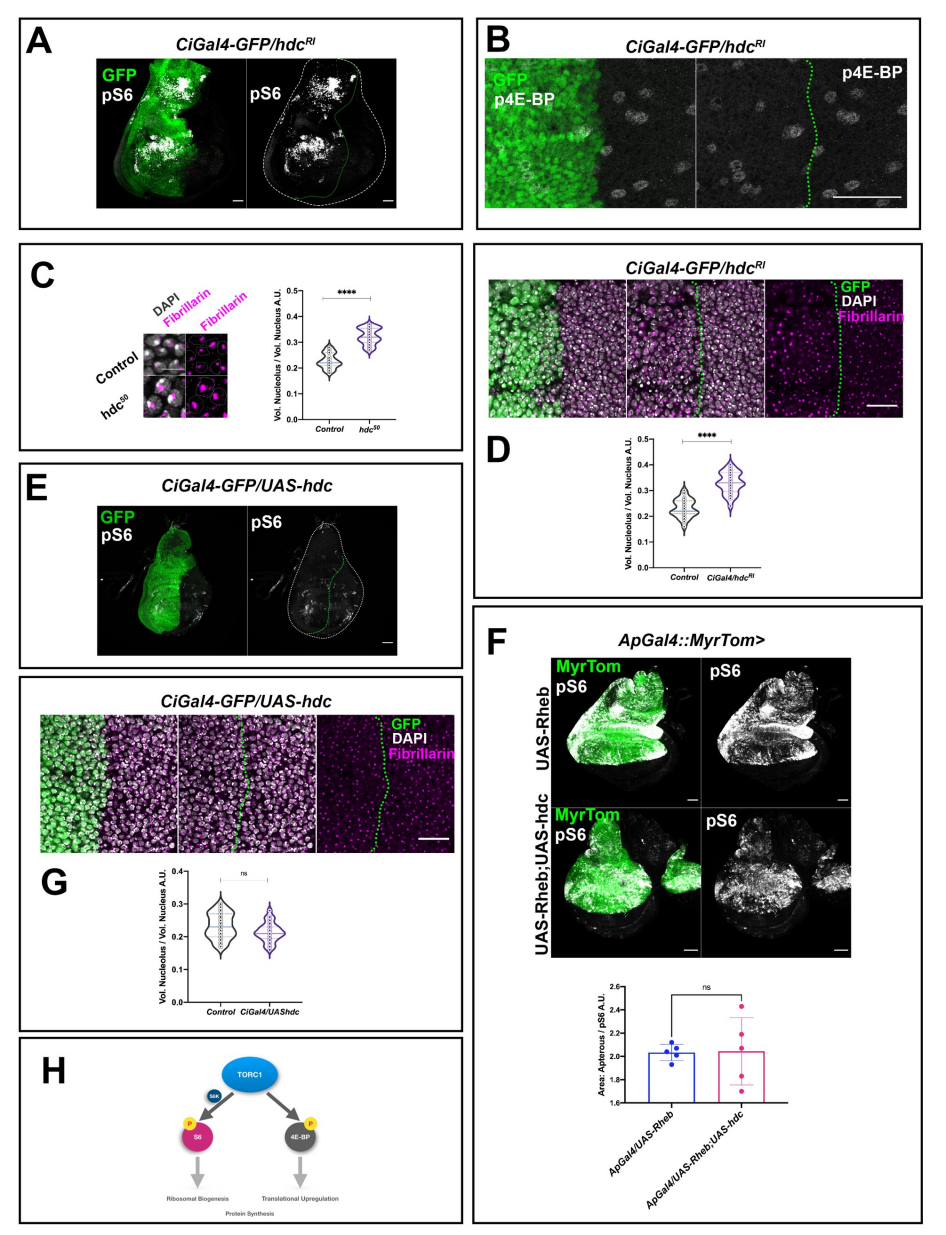
*headcase* is required for the control of TOR activity and ribosomal biogenesis. **(A)** RNAi of *hdc* induces the phosphorylation of dRp-S6 (pS6) in wing disc cells. Note the increase in pS6 signal (gray) in the anterior compartment of the wing disc where *hdc*^*RI*^ is expressed by a *Ci*Gal4 driver. Gal4 expression is labeled by UAS-GFPnls (green). Wing disc is outlined by a white dashed line and the *Ci*Gal4 area by a green dashed line. Scale bars, 40 μm **(B)** RNAi of *hdc* does not affect the phosphorylation of 4E-BP in wing disc cells. p4E-BP signal (gray) in the anterior compartment where *hdc*RNAi is expressed by a *Ci*Gal4 driver compared to the posterior counterpart of the wing disc used as control. Scale bars, 40 μm. **(C)** Nuclei of wing disc cells from control (*w*^*1118*^) and hdc mutants (*hdc*^*50/50*^) labeled by Fibrillarin staining (magenta) and DAPI (white). Scale bars, 10 μm. Violin plots show the distribution of the values of the nucleolar volume/ total nuclear volume ratio in control (*w*^*1118*^) and *hdc* mutants (*hdc*^*50/50*^). Quantification reveals the increase in nucleolar size of *hdc* mutant cells (n = 25 cells, 5 individuals per group). Unpaired t-test, Welch’s correction (**** *p<0*.*0001*). Interquartile range and mean are indicated by dashed lines. (**D**) RNAi of *hdc* induces an increase in the nucleolar size of wing disc cells. Fibrillarin staining (magenta) occupies a larger area of the nucleus in cells of the anterior compartment of the wing disc where *hdc*^*RI*^ is expressed by a *Ci*Gal4 driver, compared to the posterior counterpart of the disc. Gal4 expression is labeled by UAS-GFP (green). Nuclear DNA is stained by DAPI (white). The *Ci*Gal4 area is outlined by a green dashed line. Scale bars, 20 μm. Violin plots show the distribution of the values of the nucleolar volume/ total nuclear volume ratio in control (*CiGal4/hdc*^*RI*^ wing disc posterior compartment cells; n = 25 cells, 5 individuals) and *hdc* knockdown (*CiGal4/hdc*^*RI*^ wing disc anterior compartment cells; n = 25 cells, 5 individuals), the quantification reveals the increase of the nucleolar size in *hdc* knockdown cells. Unpaired t-test, Welch’s correction (**** *p<0*.*0001*). Interquartile range and mean are indicated by dashed lines. **(E)** Overexpression of *hdc* is not able to suppress the pS6 levels in wing disc cells. The pS6 signal levels (gray) show no observable modifications between the anterior compartment where UAS-*hdc* is expressed by a *Ci*Gal4 driver compared to the posterior counterpart of the wing disc. Wing disc is outlined by a white dashed line and the *Ci*Gal4 area by a green dashed line. Scale bars, 40 μm **(F)** Overexpression of *hdc* is not able to suppress the pS6 induction caused by TOR overactivation, as a result of *Rheb* overexpression (Unpaired t-test, Welch’s correction (*p<0.*05*). n = 5 / group. Error bars indicate SD of means.). pS6 staining (gray) is shown in L3 wing discs where UAS-*Rheb* is driven by the expression of an *ap*Gal4 (upper panels) alone, or together with a UAS-*hdc* construct (lower panels). The *apterous* Gal4 region is labeled by Myristoylated Tomato (green). Scale bars, 40 μm. **(G)** Overexpression of *hdc* does not affect the nucleolar size of wing disc cells. Fibrillarin staining (magenta) of the nucleus in cells of the anterior compartment of the wing disc where *UAS-hdc* is expressed by a *Ci*Gal4 driver, compared to the posterior counterpart of the disc does not show observable differences. Gal4 expression is labeled by UAS-GFP (green). Nuclear DNA is stained by DAPI (white). The *Ci*Gal4 area is outlined by a green dashed line. Scale bars, 20 μm. Violin plots show the distribution of the values of the nucleolar volume/ total nuclear volume ratio in control (*CiGal4/UAS-hdc* wing disc posterior compartment cells; n = 25 cells, 5 individuals) and *hdc* overexpression (*CiGal4/UAS-hdc* wing disc anterior compartment cells; n = 25 cells, 5 individuals), the analysis does not show statistically significant differences. Unpaired t-test, Welch’s correction (*p = 0*.*06*). Interquartile range and mean are indicated by dashed lines. **(H)** TORC1 (target of rapamycin complex 1) plays a key role in the regulation of cell growth and size while a wide range of signals, nutritional cues etc. is known to activate TORC1. On one hand, activation of the TOR pathway results in 4E-BP phosphorylation. This prevents 4E-BP binding to eIF4E, thus upregulating translation. On the other hand, TORC1 acts on its direct phosphorylation target, S6K, which in turn phosphorylates and activates the dRpS6 (S6) protein to shift the metabolic status of the cell towards anabolic procedures.

Previous screenings identified the Hdc protein as an interactor of the TOR pathway and showed this interaction to be relevant in response to nutrient restriction [6,9,23]. Thus, we studied whether the wing disc phenotypes caused by the loss of *hdc* in non-stress conditions during normal development are also caused by an effect of *hdc* on the TOR pathway. To this end, we examined the distribution of the phosphorylated form of dRpS6 (pS6) and p4E-BP, two readouts of TORC1 activity in *Drosophila* [24]. While we found enhanced pS6 staining in regions where we expressed the *hdc*^*RI*^ RNAi (Fig 4A), we did not observe an enhancement of the signal for p4E-BP (Fig 4B), although we cannot exclude the possibility that our failure to detect enhancement of the p4E-BP signal may emanate from the fact that 4E-BP in this tissue is expressed in very low levels [25,26]. These results indicate that the downregulation of *hdc* results in the hyperactivation of the TOR pathway in non-stress conditions during normal development, preferentially through the phosphorylation of S6 downstream of TORC1. We also examined whether *hdc* loss in non-stress conditions during normal development is associated with anabolic changes related to TOR hyperactivation that could also account, at least in part, for the overgrowth phenotypes described above. To this end, we used the fibrillarin antibody, a marker that allowed us to measure nucleolar volume [27], as the nucleolus size positively correlates with rRNA synthesis, high levels of ribogenesis, and consequently protein synthesis [28–30]. In agreement with an effect on TORC1 hyperactivation, we found larger nucleoli compared to the total nuclear volume in the wing disc cells of *hdc*^*50*^ mutants (*p<0*.*0001*) or upon *hdc*^*RI*^ expression (*p<0*.*0001*) compared to their respective controls (Fig 4C). However, while *hdc* triggered hyperactivation of the TOR pathway, we found that *hdc* overexpression did not abolish TOR pathway activity, as assayed by the levels of pS6, which did not show any observable differences to those of wild-type counterparts (Fig 4E). This finding held true even in a TOR hyperactive background, as generated by the expression of a *UASRheb* construct; the higher levels of pS6 induced by *Rheb* expression did not change significantly upon *hdc* overexpression (Fig 4F). Similarly, *hdc* overexpression did not appear to affect nucleolar size either, as the ratio of the nucleolar to the total nuclear volume was not significantly different (*p = 0*.*06*) compared to wild-type cells of the same discs (Fig 4G). Thus, *hdc* is necessary for the downregulation of TOR but is not sufficient for the complete suppression of this pathway.

**Fig 5:**
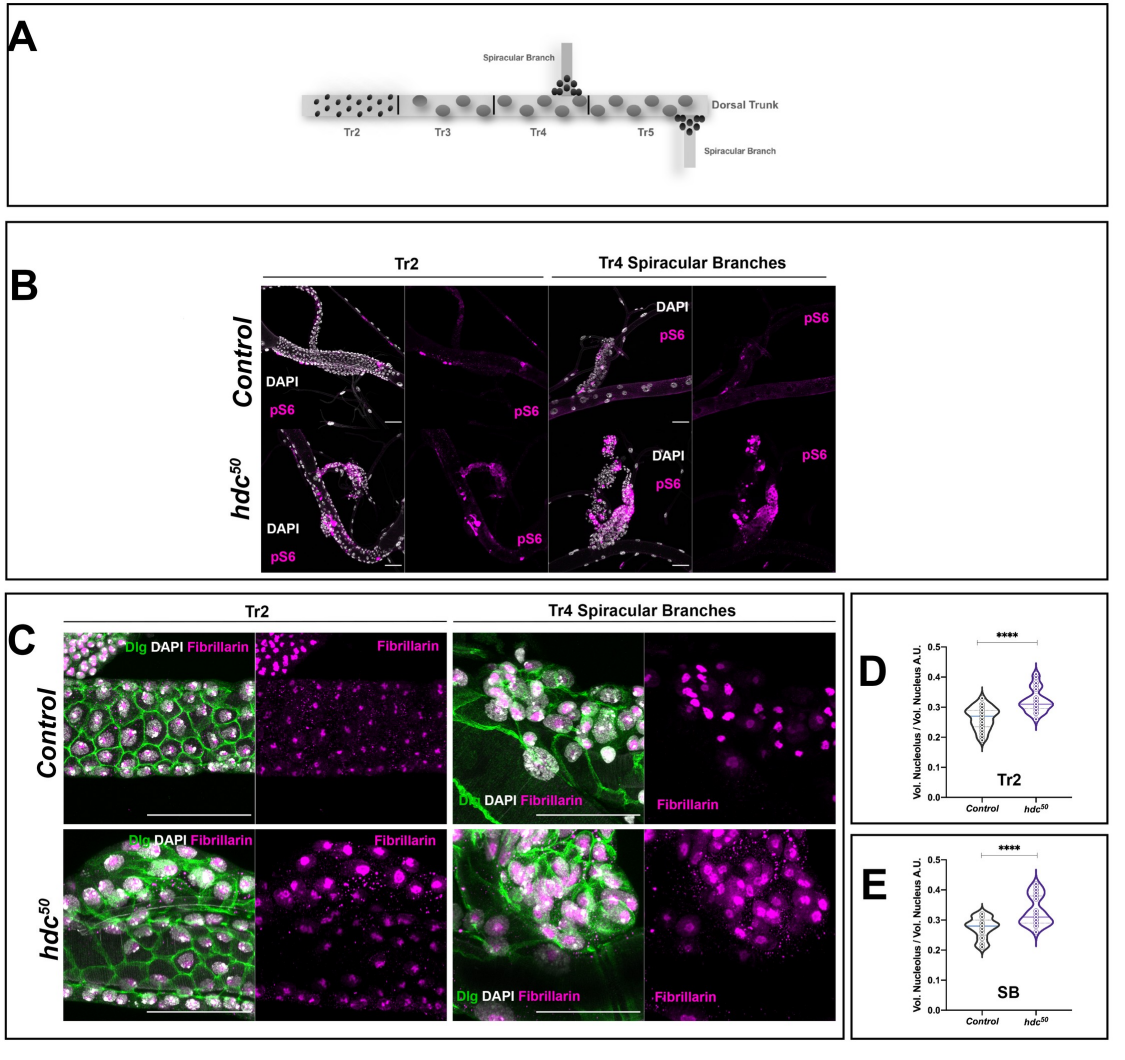
*headcase* shows similar mechanisms of action in different APC clusters. **(A)** The dorsal trunk of larval trachea is divided into 10 metameres (Tr1-Tr10). During the L3 stage, while already forming part of a functional organ, cells of the Tr2 resume proliferation and are thought to survive metamorphosis and give rise to adult tracheal structures. These particular APCs express *hdc* and are known as facultative stem cells. Tr1 and Tr3 to Tr10 cells enter endoreplication. In addition to Tr2 cells, another type of APC is found in the larval trachea. The cells of the Spiracular Branches (SB) (shown here at Tr4 and Tr5) actively divide during L3 stages and also contribute to the adult tracheal system that is formed during metamorphosis. **(B)** Loss of *hdc* induces phosphorylation of dRp-S6 (pS6) in tracheal populations of APCs. Note the increase in pS6 signal (magenta) in mutant (*hdc*^*50*^) larval tracheas compared to the controls (*w*^*1118*^) both in the Tr2 region of the dorsal trunk and the SB cells. Cell nuclei are stained by DAPI. Scale bars, 40 μm. **(C)** The nucleolar volume of *hdc* mutant (*hdc*^*50*^) tracheal APCs is larger than that of the controls. Cell membranes are labeled by Dlg (green), nuclear DNA by DAPI (white) and nucleolus by Fibrillarin (magenta). Left panel group of images show Tr2 regions of control (*w*^*1118*^) and hdc mutant (*hdc*^*50*^); right panels show SB cells of the Tr4. **(D and E)** Violin plots show the distribution of the values of the ratios for the nucleolar volume/ total nuclear volume in tracheal cells from control (*w*^*1118*^) and hdc mutants (*hdc*^*50/50*^) (n = 25 cells, 5 individuals per group), revealing the nucleolar size increase in hdc mutant cells of both the Tr2 metamere **(D)** and the SB **(E)** cells. Unpaired t-test, Welch’s correction (****p<0.*0001*). Interquartile range and mean are represented by dashed lines.

The analyses and the results reported so far refer to the wing disc cells. Although this cell population is an extensively used model of *Drosophila* APCs, the results might unveil, at least in part, specific features of wing disc cells rather than common features of APCs. To find out what might be common to the progenitor cells, we performed a similar analysis in two populations of APCs in the trachea, which is the insect respiratory system. In this regard, we examined the cells of the spiracular branches and the second tracheal metamere (Tr2) (see also Fig 5A), which have been described as dedicated and facultative stem cells respectively and that also express *hdc* [1,31]. Similar to the wing disc cells, tracheal Tr2 and spiracular cells showed enhanced pS6 staining in *hdc*^*50*^ mutants (Fig 5B). Likewise, under these circumstances, the nucleoli were larger in relation to the total nuclear volume when compared to the respective controls (*p<0*.*0001*) (Fig 5C and 5E), an observation also compatible with TORC1 hyperactivation. Thus, loss of *hdc* appears to affect the TOR pathway in a similar way in different clusters of APCs.

### hdc loss in APCs induces cell stress responses

**Fig 6:**
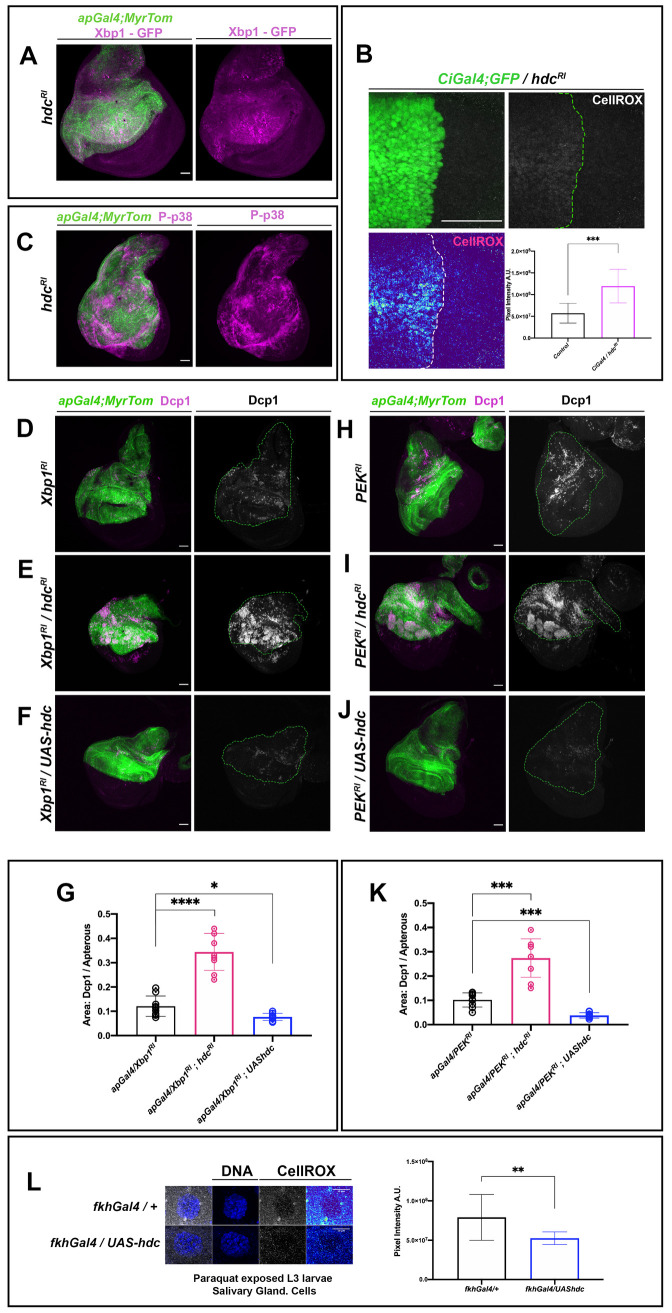
Cell stress, the UPR and *headcase*. **(A)** RNAi of *hdc* induces the splicing of Xpb1 and the nuclear localization of the latter, as a mediator of the UPR effects. Xbp1-GFP reporter (magenta) shows strong nuclear localization in the *apterous* area of the wing disc where *hdc*^*RI*^ is expressed. The *apterous* Gal4 region is labeled by Myristoylated Tomato (green). Scale bars, 40 μm **(B)** RNAi of *hdc* induces the production of ROS in wing disc cells. Note the increase in ROS signal (Gray in upper right, high ROS Red-Yellow / low ROS Cyan-Blue in the Thermal LUT in lower left image) in the anterior compartment of the wing disc where *hdc*^*RI*^ is expressed by a *Ci*Gal4 driver. Gal4 expression is labeled by UAS-GFP (green). Quantification of ROS production in the posterior wing compartment where *hdc*^*RI*^ is expressed in comparison to the posterior wild-type shows increased ROS upon RNAi of *hdc* and results from 2 ROIs in Sum. Intensity stacks per compartment of the wing disc, from the wing discs of 5 individuals. Unpaired t-test, Welch’s correction (****p<0.*0001*). Error bars represent SD of means. Scale bars, 40 μm. **(C)** RNAi of *hdc* induces the phosphorylation of p38 (P-p38 in magenta) in the *apterous* area of the wing disc where *hdc*^*RI*^ is expressed. The *apterous* Gal4 region is labeled by Myristoylated Tomato (green). Scale bars, 40μm **(D, E, F, G, H, I, J and K)** Synchronous expression of RNAi for *hdc* and *Xbp1* **(E),** as well as for *hdc* and *PERK* **(I),** results in the observed increase in apoptotic death, compared to the UPR disruption after knockdown of its mediators (*Xbp1* **(D)** and *PERK* **(H)**) alone. Synchronous overexpression of a *UAS-hdc* with an RNAi against Xbp1 **(F)** and PEK **(J)** results in the decrease in the observed apoptotic death, compared to the UPR disruption after knockdown of its mediators (*Xbp1* **(D)** and *PERK* **(H)**) alone. Dcp1 apoptotic marker is labeled in magenta (left panels) and white (right panels) of each set. The *apterous* Gal4 region is labeled by Myristoylated Tomato (green) and outlined by a dashed green line in grey colored Dcp1 images (right panels of each set). Apoptotic cell death increase in *hdc*^*RI*^*;Xbp1-RNAi* and *hdc*^*RI*^*;PEK-RNAi* double RNAis and decrease in *UAS-hdc;Xpb1RNAi* and *UAS-hdc;PEKRNAi* were calculated by comparison of the ratios of Dcp1-positive area within the Gal4-expressing area of each wing disc to the same ratio resulting from *Xbp1*RNAi **(G)** and *PEK*RNAi **(K)** wing discs respectively (n = 9 wing discs per genotype). Unpaired t-tests, Welch’s correction (*p<0.05; **p<0.01; ***p<0.001; ****p<0.*0001*) Scale bars, 40 μm **(L)** Ectopic expression of *hdc* reduces the production of ROS in salivary gland cells of L3 larvae exposed to paraquat. Note the decrease in ROS signal (high ROS Red-Yellow / low ROS Cyan-Blue in the Thermal LUT in right upper and lower image) in a cell of a salivary gland where *hdc* is ectopically expressed by a *fkh*Gal4 driver (*fkhGal4/UAS-hdc*), compared to the respective control (*fkhGal4/+*). DAPI stain marks nuclear DNA (Blue), ROS stained by the CellROX Deep Red reagent is shown in gray and thermal LUTs. Quantification of ROS production per genotype results from 3 ROIs of Sum. Intensity stacks from the salivary glands of 5 individuals. Unpaired t-test, Welch’s correction (**p<0.*01*). Error bars represent SD of means. Scale bars, 10 μm.

Since *hdc* loss in APCs caused hyperactivation of the TOR pathway and an increase in ribogenesis, it may also lead to a high increase in cell protein load. In fact, such an increase in protein load may cause ER stress and the induction of the protective Unfolded Protein Response (UPR) mechanism [32]. Thus, to examine whether the UPR is activated in *hdc* mutant cells, we used an *in vivo* UPR marker, the *xbp1-EGFP* construct, which generates an *xbp1* spliced isoform that accumulates in the nucleus in case of UPR induction [33]. Accordingly, we observed a higher nuclear localization of GFP in the wing disc when the *UAS-xbp1-EGFP* construct was expressed upon *hdc* RNAi *(hdc*^*RI*^) (Fig 6A).

To further confirm cell stress upon *hdc* loss, we resorted to the CellROX Oxidative Stress Reagents, which emit a strong fluorogenic signal upon oxidation and are commonly used to detect Reactive Oxygen Species (ROS). An enhanced CellROX signal was observed in a wing region upon *hdc* RNAi induction compared to the rest of the disc (Fig 6B). Likewise, the Hsp70-GFP marker sensing acute stress conditions [34] produced a stronger GFP signal in *hdc* RNAi wing discs compared to discs from control larvae (S3A Fig). Finally, the phosphorylated p38 Stress-Activated Protein Kinase (P-p38) [35–38] was strongly activated in wing discs upon *hdc* RNAi (Fig 6C).

The prolonged activation of the UPR and/or its failure to restore the normal function of the ER may lead to apoptosis, which could account for the above reported cell death upon *hdc* loss. Apoptosis triggered by ER stress is mediated mainly by the activity of the c-Jun N-terminal kinase (JNK) pathway [38,39], which can be detected by a *puc*- lac-Z reporter, which is both a downstream effector and a negative regulator of JNK [40,41]. In agreement with activation of the JNK pathway upon *hdc* loss, we observed *puc*-lac-Z staining in the wing disc region subjected to *hdc* RNAi (S3B Fig). Finally, we examined whether ROS may be the cause for the apoptosis upon depletion of *hdc*. To this end, we induced enzymatic depletion of O_2_^-^ and H_2_O_2_ by overexpression of *Catalase* and *SOD1*, together with *hdc* RNAi, but overexpression of *Catalase* and *SOD1* failed to rescue the apoptosis induced by the *hdc* RNAi. This observation indicates that ROS are most probably produced as a result of the tissue damage caused by loss of *hdc* and probably are not triggering apoptosis (S3C and S3C’ Fig).

### hdc as a cell stress protective factor

The above results suggest that *hdc* may protect APCs from the deleterious effects of ER-stress. Indeed, malfunction of the UPR is one of the conditions leading cells into stress. Thus, to assess the putative protective role of *hdc*, we used the *ap*Gal4 construct to drive the expression of the RNAi of two genes that mediate the effects of the UPR through its major branches in *Drosophila* [42], namely *xbp1* (Fig 6D) and *PERK* (Fig 6H), and compared their effect in the presence of a normal dose of the *hdc* gene or upon *hdc* knockdown by means of RNAi (*hdc*^*RI*^). In each case, we measured the area of the of Dcp1-positive cells and used the magnitude of the ratio of Dcp1/*ap*Gal4 area as an estimation of the severity of apoptotic cell damage induced by the RNAis. In fact, cell death in the wing discs increased upon *xbp1* ^*RI*^ or *PERK* ^*RI*^, while the levels of Dcp1 staining in the double knockdown of *hdc*^*RI*^ and *xbp1*^*RI*^ (Fig 6E) or *hdc*^*RI*^ and *PERK*^*RI*^ (Fig 6I) were higher than those of *xbp1*^*RI*^ (*p<0*.*0001*) (Fig 4G) or *PERK*^*RI*^ (*p<0*.*0001*) (Fig 6K) alone, respectively. These results indicate that *hdc* activity attenuates the deleterious effects of a defective cell stress response.

Interestingly, we also found that *hdc* overexpression reduced the wing disc levels of Dcp1 signal due to knockdown of either *xbp1*^*RI*^ (*p<0*.*05*) or *PERK*^*RI*^ (*p<0*.*01*) (Fig 6F and 6J). This observation suggests that overexpression of *hdc* decreases the ER-stress levels of UPR-defective cells, resulting in the reduction of the apoptosis caused by UPR deregulation (Fig 6G and 6K). Additionally, to examine whether *hdc* might provide a protective role in stressed cells, we used a *nubGal4* construct to drive *hdc*^RI^ in the wing disc, and we exposed L3 larvae to an oxidative stress inducer for 12h by feeding them with fly food containing paraquat (20μg/ml). Loss of *hdc* in stress conditions resulted in a further increase in the levels of apoptosis, as detected by the Dcp1 marker (S3D and S3D’ Fig), thereby indicating the protective role of this gene. Furthermore, to examine a similar role of *hdc* in cells in which it is not normally expressed, we repeated the paraquat exposure experiment using a *fkhGal4* driver to ectopically express *hdc* in cells of the salivary glands. In that case, we observed a significant reduction in ROS production in the case of *fkhGal4/UAShdc* compared to the control (*p<0*.*05*) (Fig 6L), thereby indicating that *hdc* can act protectively against ROS induction in tissues undergoing oxidative stress.

## Discussion

Progenitor cells, which are defined by their capacity to self-renew and their potential to differentiate, have a different developmental program compared to their differentiated cell counterparts, including distinct patterns of cell proliferation, particular metabolic features, distinct responses to the systemic hormone signaling and reactions to internal and external stimuli. In *Drosophila*, as in many other insects, a key population of progenitor cells is found in larvae, and upon metamorphosis, these give rise to the variety of cells in the adult organism. It has been known for quite some time that *Drosophila* APCs distinctly express *hdc* [5]. While *hdc* has been previously described as a nutrient-restriction (NR) specific growth regulator [6], its role in normal development remained largely unknown.

### A systemic and a local role for headcase

Here we have identified a dual role for *hdc*. On the one hand, through its function in the prothoracic gland, the ecdysone-producing organ, *hdc* is required for the proper synthesis and/or delivery of this hormone. Ecdysone is the molting hormone in insects and it governs the growth of imaginal discs (clusters of APCs), larval molting and entry into metamorphosis. Thus, by controlling ecdysone, *hdc* has a systemic role on APCs, promoting their growth and differentiation. On the other hand, we have characterized the local role of *hdc* in APCs, particularly in the imaginal wing disc as a prototypical model for *Drosophila* APCs, where it is necessary for their survival and limits their uncontrolled proliferation. Therefore, the phenotype of the APCs in the *hdc* mutants is due to the combined effect of impairing both its systemic role and its local requirement. Actually, the systemic and local requirements identified here, help to better understand the phenotype of the *hdc* mutants. In addition to the lethality of *hdc* mutants in pupal stages previously published [5], we report that lethality occurs throughout development and that only some individuals reach the pupal stage. Lethality during early developmental stages is probably mainly due to the effect of *hdc* on ecdysone production and/or delivery. Of note, impairment of the systemic or the local requirements of *hdc* has opposing effects. This observation suggests that the local effect of *hdc* on APCs tends to confer protection against or minimize the consequences of its systemic impact.

### Interaction of hdc with the TOR pathway

The phenotype caused by the downregulation of *hdc* in the APCs seems to result from the disturbance of distinct molecular mechanisms and cannot be attributed solely to a general failure of cell metabolism. Several observations support this notion. First, our results indicate a clear relation between the *hdc* phenotype and the activity of the TOR pathway. Second, they reveal that the interaction of *hdc* phenotypes and the TOR pathway is mediated mainly through its dRpS6 branch and no obvious changes in the phosphorylation status of 4E-BP in another branch of the TOR pathway are observed, pointing again to a rather specific alteration. Finally, previous reports established a physical interaction of Hdc protein with another component of TOR signaling, namely Unkempt [6,23]. Nevertheless, we think that the interaction of *hdc* with the TOR pathway does not account for all the functions of *hdc* since our own observations point also to an interaction with members of the heat shock machinery. Clearly, further studies are therefore needed to provide an understanding of the global function of *hdc* and above all to determine the nature of the Hdc protein, which has remained elusive up to now.

### Hdc as a stress protector

Our results show that APCs require *hdc* function to avoid entering into stress. Moreover, forced expression of *hdc* can also provide a stress protective effect on non-progenitor cells, which are usually devoid of *hdc* expression. The role of *hdc* as a stress protector is consistent with previous analyses showing a growth-suppressing role for this gene in conditions of nutrient restriction (NR), where low metabolic activity has to be sustained. Indeed, we consider that the effects of *hdc* observed previously in stress conditions are merely an exacerbated case of its role in normal development. In fact, in non-stress conditions, we show a measurable effect of *hdc* loss on tissue growth, together with a detrimental effect on tissue survival, not reported previously [6] and that was at odds with the indispensable role of *hdc* during development. The difference between our analysis and others may lie in the genetic tools we employed, as the RNAi against *hdc* used here knocks down the transcript levels of the gene more efficiently (see also Materials and Methods and S2 Fig). During development, various cellular mechanisms, including the TOR and FOXO pathways and also the UPR, respond both to normal developmental cues and stress stimuli to ensure the maintenance of cellular and organismal homeostasis [43–47]. We therefore think that several molecular components, including *hdc*, can be employed to ensure the differential cellular response and/or the magnitude of the activity of the aforementioned mechanisms upon different stimuli, specifically in APCs.

### Stress and metamorphosis

Given the specific expression of *hdc* in APCs, it is of particular interest to identify it also as a factor that regulates the levels of ecdysone reaching this cell population. In fact, what might be the functional relevance of having the same factor conferring APCs with a protective shield against stress and also regulating ecdysone? Considering our results, we would like to propose a novel view regarding cell responses to ecdysone activity during development. In particular, ecdysone plays a key role in regulating metamorphosis, which, while a customary event at the organism level, may be considered highly stressful at the cellular level. Indeed, metamorphosis has devastating effects for the majority of larval cells, which die at metamorphosis, while only the APCs are able to survive [48]. Thus, as ecdysone is required to promote both larval cell death and APC proliferation and differentiation into adult structures, we favor the notion that *hdc* is part of the mechanism that allows APCs to respond differentially and to countervail the ecdysone levels that are required for the specific cell responses during the transition between developmental stages. Moreover, the overall conditions of APCs differ from those of larval cells not only at metamorphosis but also during embryonic and larval stages. For example, the growth rates of APCs and larval cells are suggested to be different [49,50]. Actually, growth rates are clearly influenced by ecdysone signaling [18,20], as is also the case for the metabolic shift of stem cells from glycolysis to oxidative phosphorylation, the production of oxidative agents [51] and the susceptibility to external stress stimuli [52]. Further studies will be required to determine whether these other properties of APCs are also under the influence of *hdc* activity.

### hdc, human heca, stress and tumorigenesis

Hdc is the founding member of a group of homolog proteins identified from *C*. *elegans* to humans. In humans, the Hdc homolog, termed Heca, has been found associated with different kinds of cancers but its function has not yet been identified and its role remains controversial. However, similar to the *Drosophila* homolog, Heca physically interacts with components of mTORC1 [6]. In addition, the finding that the *Drosophila* homolog *hdc* is specifically expressed in progenitor cells and that it confers stress protection opens up a new way to be explored regarding the role of the human Heca and its contribution to carcinogenesis. First, research has established that ER and oxidative stress promote and contribute to initiation and progression of tumorigenesis (for reviews, see [53,54]). And second, Heca is downregulated in different kind of cancerous tissues, which has led to propose that it acts as a tumour suppressor [55–57]. All together, we would like to introduce the hypothesis that, similarly to Hdc, Heca could act as a stress protector and its downregulation may induce stress conditions favouring the initiation and progression of tumorigenesis.

## Materials and methods

### Fly strains and genetics

All fly stocks were reared at 25°C on standard flour/agar *Drosophila* media. The Gal4/UAS system [58] was used to drive the expression of transgenes at 29°C. The following strains were provided by the Bloomington Drosophila Stock Center (BDSC) or the Vienna Drosophila RNAi Center (VDRC): *Df(3R)BSC503 (BDSC 25007)*; *Df(3R)ED6332 (BDSC 24141)*; *hdc*^*43*^ *(BDSC 64063)*; *hdc*^*50*^ *(BDSC 64064)*; *hdc*^*BG23007*^ *(BDSC 12410)*; *UAS-hdc (BDSC 64056)*; *hdc*^*RI*^ *(VDRC 104322)*; *hdc*^*R2*^ *(VDRC 45069)*; *hdc*^*R3*^ *(BDSC 30489)*; *UAS-p35 (BDSC 5073)*; *UAS-Rheb (BDSC 9689)*; *UAS-Xbp1-EGFP (BDSC 60731)*; *Xbp1*^*RI*^ *(BDSC 36755)*; *PEK*^*RI*^ *(VDRC 110278)*; *UAS-myristoylated-Tomato (BDSC 32222)*; *UAS-CD8-GFP (BDSC 5137)*; *UAS-src-GFP (BDSC 5429)*; *UAS-myrRFP (BDSC 7138)*; *hsp70-GFP (BDSC 51354)*; *phmGal4 (BDSC 80577)*; *fkhGal4 (BDSC 78060)*; *apGal4 (BDSC 3041)*, *UAS-GFPnls (BDSC 4776); TM3-cherry (BDSC 35524); puc-LacZ (BDSC 11173);* The following strains are described in Flybase: *nub-Gal4* [59]*; Ci-Gal4* [60]; *sal*^*E/PV*^ [61]; *amn*^*c651*^ [10]. Gal4 drivers were recombined to UAS fluorescent markers described here. *UAS-SOD1*::*UAS-Cat* recombined construct was kindly provided by F. Serras. *phmGal4*::*YPetAtet* [16] was kindly provided by X. Franch-Marro. *w*^*118*^ strain was used as control.

### Immunohistochemistry

For fluorescent imaging, PGs, wing discs and tracheas from L3 larvae were dissected in 1x phosphate-buffered saline (PBS) and fixed in 4% formaldehyde for 20 min at RT. The tissues were rinsed in 0.1% Triton X-100 (PBST), blocked in PBST + BSA 0.5% for 1h and incubated at 4°C with primary antibodies diluted in PBST + BSA 0.5% overnight. After incubation with primary antibodies, the tissues were washed with PBST (3 x 10min washes) and incubated with the corresponding secondary antibodies (Alexa Conjugated dyes 488, 555, 647, Life Technologies, 1:400) for 2 h at RT, followed by 3 x 10min washes with PBST, and then rinsed with PBS before mounting. The following primary antibodies were used: anti-HDC, U33, Developmental Studies Hybridoma Bank (1:3); anti-Dcp1 [Cell Signaling, Asp216 #9578] (1:100); anti-phospho-4EBP [Cell Signaling, (Thr37/46), #2855] (1:100); anti-Fibrillarin [Abcam, ab5821] (1:500); anti-phospho-RpS6, kindly provided by A. Teleman [24] (1:200); anti-phospho-p38 [Cell Signaling, (Thr180/Tyr182), (3D7) #9215] (1:50); anti-Dlg, 4-F3, Developmental Studies Hybridoma Bank (1:100); anti-βGalactosidase, 40-1a, Developmental Studies Hybridoma Bank (1:200); anti-GFP, Tebu-bio Rockland (1:500). The tissues were mounted in Vectashield medium with DAPI (Vector Laboratories, H1200).

### Death scoring and timed sample collections

*hdc* mutant alleles were balanced with fluorescent *TM3-cherry* chromosome to select against the presence of fluorescence the homozygous mutants during embryonic and early larval stages. For death scoring during development, flies were allowed to lay eggs on agar plates supplied with fresh yeast paste for 2–3 h and 15–30 embryos were collected and split into plates containing standard fly food. The embryos were scored for genotype and monitored throughout development at 25°C in time intervals of 1–5 h during the daytime, while death events were scored till pupal stages. For pupariation timing, larvae were synchronized by allowing flies to lay eggs on agar plates supplied with fresh yeast paste for 2–3 h, and 20–30 freshly eclosed L1 per genotype were incubated at 25°C until they reach the pupal stage. Pupariation time was scored every 3–4 h during the daytime. The data obtained were ordered by time and cumulative percentages of pupariation and were analyzed in GraphPad Prism 8.4.3 Software.

### 20E rescue experiments

20E (Sigma, H5142) was dissolved in ethanol at 5 mg/ml. Standard fly food was supplemented with 0.5 mg/ml of 20E or an equal amount of ethanol (control). For rescue experiments, L3 larvae were collected and reared on 20E-supplemented medium or control medium with ethanol.

### ROS detection

To detect ROS, we used the CellROX Deep Red Reagent (Life Technologies), an indicator of oxidative stress in living cells. Tissues were dissected in Schneider’s medium and incubated in 5 μΜ of CellROX in Schneider’s medium at 25°C for 20 min. The tissues were then rinsed with 1xPBS, fixed in 4% formaldehyde for 15 min and mounted in Vectashield medium with DAPI (Vector Laboratories, H1200). Confocal microscopy for the detection of fluorescence was performed within a maximum of 2 h after fixation to avoid signal loss.

### Oxidative stress induction

Third instar larvae were transferred to vials containing standard fly food with 20 μg/mL paraquat. To minimize loss of oxidative capacity, paraquat was added to liquid fly food at 45°C. Third instar larvae were left to feed in this medium for 12 h prior to dissection. Controls were run in parallel with larvae fed the same medium without paraquat addition.

### RNA extraction and qRT-PCR

After dissection in 1xPBS and isolation of the wing pouch region, total RNA was extracted from 10 wing discs per genotype using the Trizol reagent, further purified using RNeasy columns (Qiagen). For the quantification of ecdysone responsive genes, total RNA was extracted from whole larval extracts at 120h A.E.L. from 5 larvae per genotype. Reverse transcription was done using the High Capacity cDNA Archive Kit (Applied Biosystems). The 480 LightCycler was used for real time PCR reactions. Gene expression levels were normalized to *actin42A* and *a-tubulin* mRNA levels. The primers used in this study are listed in S1 Table.

### Imaging, acquisition and image analysis

Images were obtained with Zeiss 780 and Zeiss 880 confocal microscopes, using the LD LCI PlanApo 25x 0.8 and Plan-Apochromat 63x/1.4 Oil DIC M27 objectives. For image acquisition, XY was set to 1024x1024 and the Z path was set to optimal as defined by the Zeiss ZEN lite software. The same imaging acquisition parameters were used for all the comparative analyses. Images were processed with the Imaris Software (Oxford Instruments) and Fiji [62]. For the quantification of apoptosis, the Dcp1-positive area was defined for each territory using Fiji and was normalized to the total wing disc area or the Gal4-expressing territory measured per disc. The same procedure was followed for the quantification of pS6 positive area, normalized to the *apGal4* expressing area in *apGal4/UAS-Rheb* and *apGal4/UAS-Rheb;UAS-hdc* wing discs. Volumetric measurements were performed using the Imaris Software. Prothoracic gland cell count was done in Imaris after image segmentation based on the DAPI and GFP channels to define the cell nuclei found within the *phmGal4*-expressing areas. The C value of the same cells was also defined by an Imaris workflow. The intensity sum values of the DAPI channel for cells of the PG was normalized to the average of the intensity sum values of diploid nuclei of imaginal discs or tracheas of the same preparations. The Imaris spots function was used for the quantification of Atet vesicle structures in prothoracic glands of control and *hdc*^*RI*^ larvae. For CellROX quantification, pixel intensity in 25 μm^2^ regions of sum intensity z-stacks of same total number of slices and path, were measured for each group in Fiji. Final Figures presented in this paper were produced in Adobe Photoshop CC software. The numerical data that resulted from image analyses can be found in S1 File.

### Statistical analysis

Statistical analysis, data processing and graphical representations were performed in GraphPad Prism 8.4.3 Software. Welch’s t-tests were used to determine significant differences between two groups to correct for unequal sample distribution variance. For the comparison of more than two groups, Welch’s ANOVA was used, followed by Dunnett’s T3 post hoc tests for multiple comparisons between groups. Developmental profiles of *phmGal4/+* and *phaGal4/hdc*^*RI*^ populations were compared using Fisher’s exact test. Detailed data from the statistical analyses can be found in S1 File.

## Supporting information

S1 Fig Knockdown of *hdc* in PG results in larval lethality and L3 larval arrest. Knockdown of *hdc* in the PG results in larval arrest at the L3 stage and lethality during the L1 and L2 stages. For all the RNAi (*hdc*^*RI*^ and *hdc*^*R2*^) and Gal4 (*phm22* and *amn*^c651^) combinations tested. *amnc651* results in weaker phenotypes with both RNAis in terms of L1-L2 lethality and larval arrest at L3. 28% of *amnc651/ hdcRI* die at L1-L2 compared to 40% of *phm22/ hdc*^*RI*^, and 13% of *amn*^*c651*^*/hdc*^*R2*^ die at L1-L2 compared to a 19% of *phm22/ hdc*^*R2*^. In addition, 41% of *amn*^*c651*^*/ hdc*^*R2*^ either die at the transition of L3 to pupa or at white pupal stages compared to 11% of *phm22/ hdc*^*R2*^. In terms of RNAi efficiency, *hdcRI* shows consistently stronger phenotypes in comparison to *hdc*^*R2*^ with 100% larval arrest at L3, for both Gal4 drivers tested. Circles indicate individuals used for each experimental group (n = 100 / experimental condition).
(TIF)

S2 Fig Effects of *hdc* knockdowns in wing tissues and *hdc* RNAi efficiency. (A) Adult wing phenotypes of *hdc* knockdown in the wing pouch using the nubGal4 driver. *nubGal/hdc*^*RI*^ show a strong phenotype of severely deformed wings (100%), which is rescued with synchronous expression of a UAS-hdc construct. *nubGal/hdc*^*R2*^ show wing deformations less frequently (4%) while a third RNAi against hdc (BDSC: TRiP.HM05231, noted here as *hdc*^*R3*^) does not show any observable effect in the same test, probably due to inefficient knockdown of the gene, as shown by a higher abundance of hdc transcripts detected through qPRCs (see also S2B Fig). (B) Relative mRNA expression of the hdc gene in L3 wing discs of control and three different knockdowns with the RNAis tested in this study. *hdc*^*RI*^ results in 72% knockdown of the gene’s transcripts compared to 65% with *hdc*^*R2*^ and 32% with *hdc*^*R3*^. Normalization was done using the actin and tubulin genes as references. (C) *nubGal/hdc*^*RI*^ and *nubGal/hdc*^*R2*^ L3 wing discs stained with an antibody against Hdc. Note that both RNAis are able to downregulate the levels of the protein, as shown by reduced fluorescent signal in the pouch region of the disc.
(TIF)

S3 Fig *hdc* knockdown results in high activity of cell stress markers and JNK; apoptosis induced by *hdc* loss cannot be rescued by enzymatic depletion of free radicals. (A) Cells depleted for *hdc* activate heat shock chaperones. Hsp70 promoter shows enhanced activity in the pouch region of the wing disc of *nubGal;hsp70GFP/hdc*^*RI*^ compared to the *nubGal;hsp70GFP*/+ control, used here to monitor the endogenous activity of the promoter in wild type conditions. (B) The JNK activity marker puckered (*puc*) is shown to be activated in the apterous region of *apGal4*::*MyrTom/hdc*^*RI*^ L3 wing discs where hdc is knocked down. *Puc-LacZ* used to mark *puc* expression (magenda) and Dcp1 (blue) to mark apoptotic cells in the *apGal4* region. Scale bar, 40 μm (C, C’) Enzymatic depletion of O_2_—and H_2_O_2_ by overexpression of Catalase and SOD1, together with *hdc* RNAi, fail to rescue the apoptosis induced by the absence of *hdc*. Comparisons of the ratios for the Dcp1-positive area / total wing disc area between *sal*^*E/PV*^*/hdcRI* and *sal*^*E/PV*^*/hdc*^*RI*^; *UAS SOD1*::*UAS Cat* wing discs showed no statistically significant differences in (C’). Unpaired t-test, Welch’s correction (p = 0.51). n = 10 / group. Error bars indicate SD of means. Scale bars, 40 μm. (D,D’) hdc acts protectively against tissue damage in conditions of cell stress. Comparisons of the ratios for the Dcp1-positive area / wing disc pouch area between wing discs from *nubGal4/hdc*^*RI*^ L3 larvae fed normal food and *nubGal4/hdc*^*RI*^ L3 larvae fed the oxidative stress agent paraquat for 12 h show higher levels of apoptosis in the wing pouch area of the latter (D’). Unpaired t-test, Welch’s correction (*p<0.05). n = 5 / group. Error bars indicate SD of means. Scale bars, 40 μm.
(TIF)

S1 Table Primers used in qRT-PCR experiments. (DOCX)

S1 File Numerical data used in this study and detailed statistics. (XLSX)
