# Supplementary figures and images for: Systemic and local effect of the Drosophila headcase gene and its role in stress protection of Adult Progenitor Cells
Source: PLoS Genet. 2021 Feb 8;17(2):e1009362. doi: 10.1371/journal.pgen.1009362 (PMC7895379; doi:10.1371/journal.pgen.1009362)

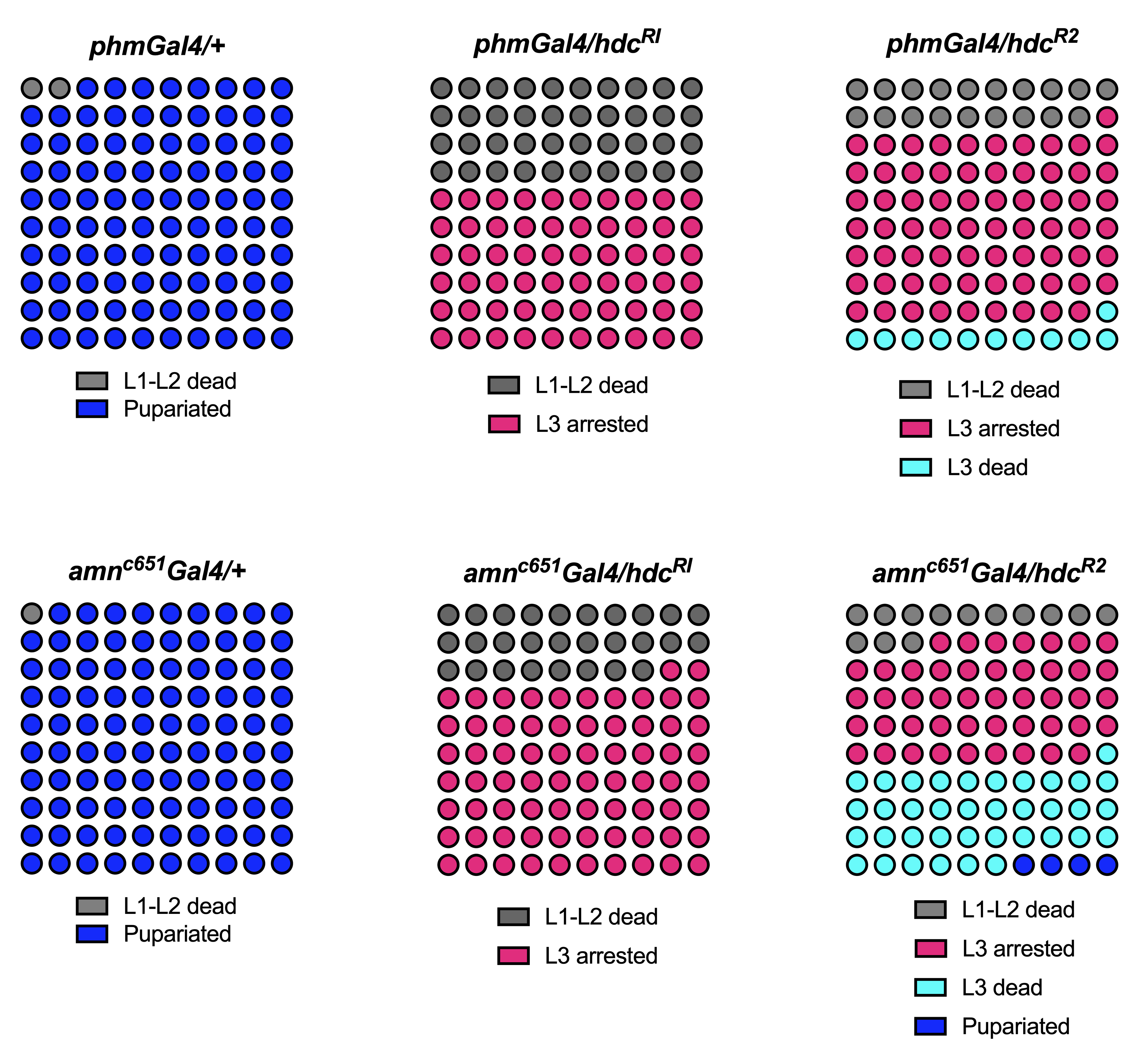

Supplement: S1 Fig — Knockdown of hdc in the PG results in larval arrest at the L3 stage and lethality during the L1 and L2 stages. For all the RNAi (hdcRI and hdcR2) and Gal4 (phm22 and amnc651) combinations tested. amnc651 results in weaker phenotypes with both RNAis in terms of L1-L2 lethality and larval arrest at L3. 28% of amnc651/ hdcRI die at L1-L2 compared to 40% of phm22/ hdcRI, and 13% of amnc651/hdcR2 die at L1-L2 compared to a 19% of phm22/ hdcR2. In addition, 41% of amnc651/ hdcR2 either die at the transition of L3 to pupa or at white pupal stages compared to 11% of phm22/ hdcR2. In terms of RNAi efficiency, hdcRI shows consistently stronger phenotypes in comparison to hdcR2 with 100% larval arrest at L3, for both Gal4 drivers tested. Circles indicate individuals used for each experimental group (n = 100 / experimental condition). (TIF) [file pgen.1009362.s001.tif]

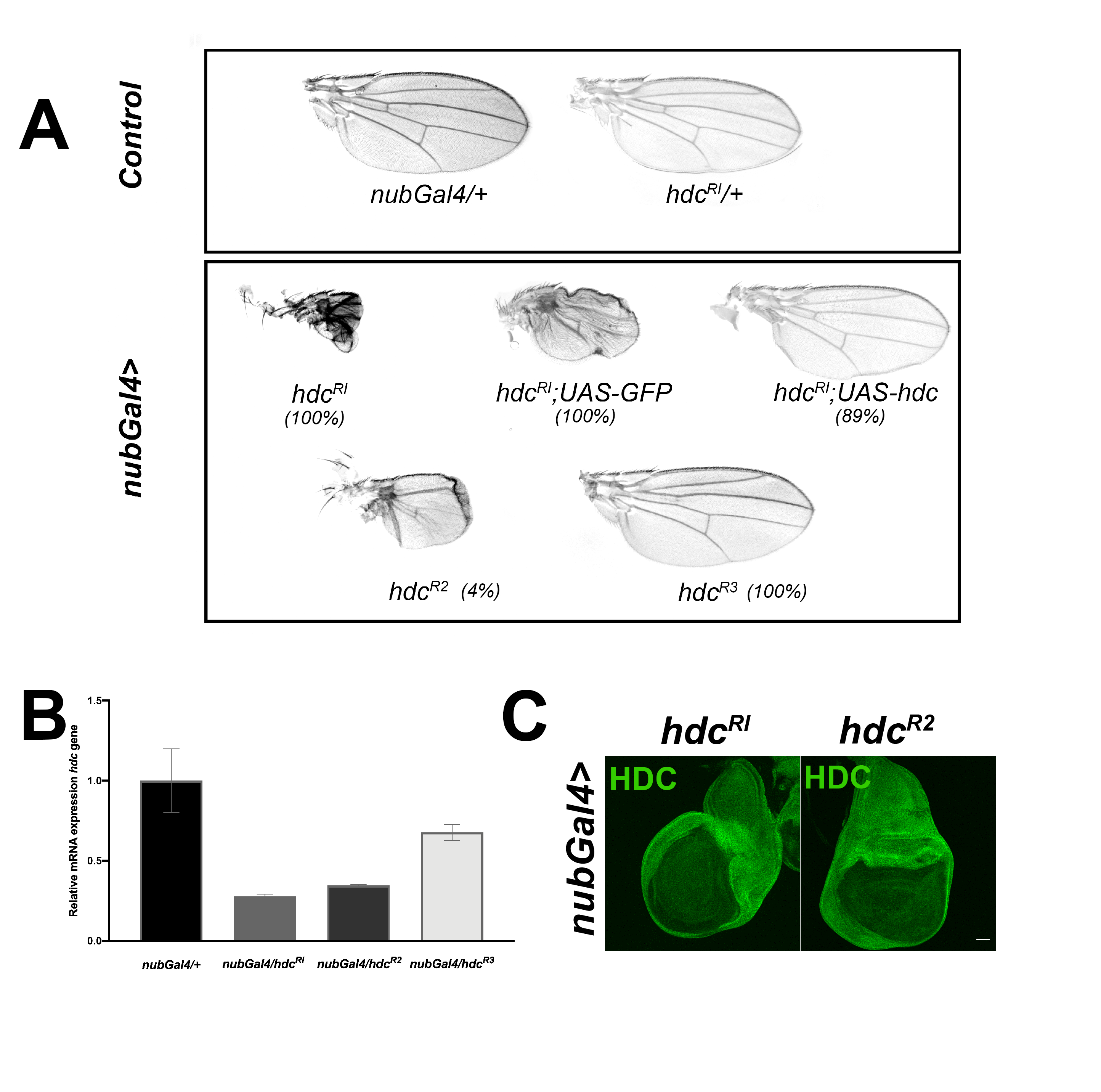

Supplement: S2 Fig — (A) Adult wing phenotypes of hdc knockdown in the wing pouch using the nubGal4 driver. nubGal/hdcRI show a strong phenotype of severely deformed wings (100%), which is rescued with synchronous expression of a UAS-hdc construct. nubGal/hdcR2 show wing deformations less frequently (4%) while a third RNAi against hdc (BDSC: TRiP.HM05231, noted here as hdcR3) does not show any observable effect in the same test, probably due to inefficient knockdown of the gene, as shown by a higher abundance of hdc transcripts detected through qPRCs (see also S2B Fig). (B) Relative mRNA expression of the hdc gene in L3 wing discs of control and three different knockdowns with the RNAis tested in this study. hdcRI results in 72% knockdown of the gene’s transcripts compared to 65% with hdcR2 and 32% with hdcR3. Normalization was done using the actin and tubulin genes as references. (C) nubGal/hdcRI and nubGal/hdcR2 L3 wing discs stained with an antibody against Hdc. Note that both RNAis are able to downregulate the levels of the protein, as shown by reduced fluorescent signal in the pouch region of the disc. (TIF) [file pgen.1009362.s002.tif]

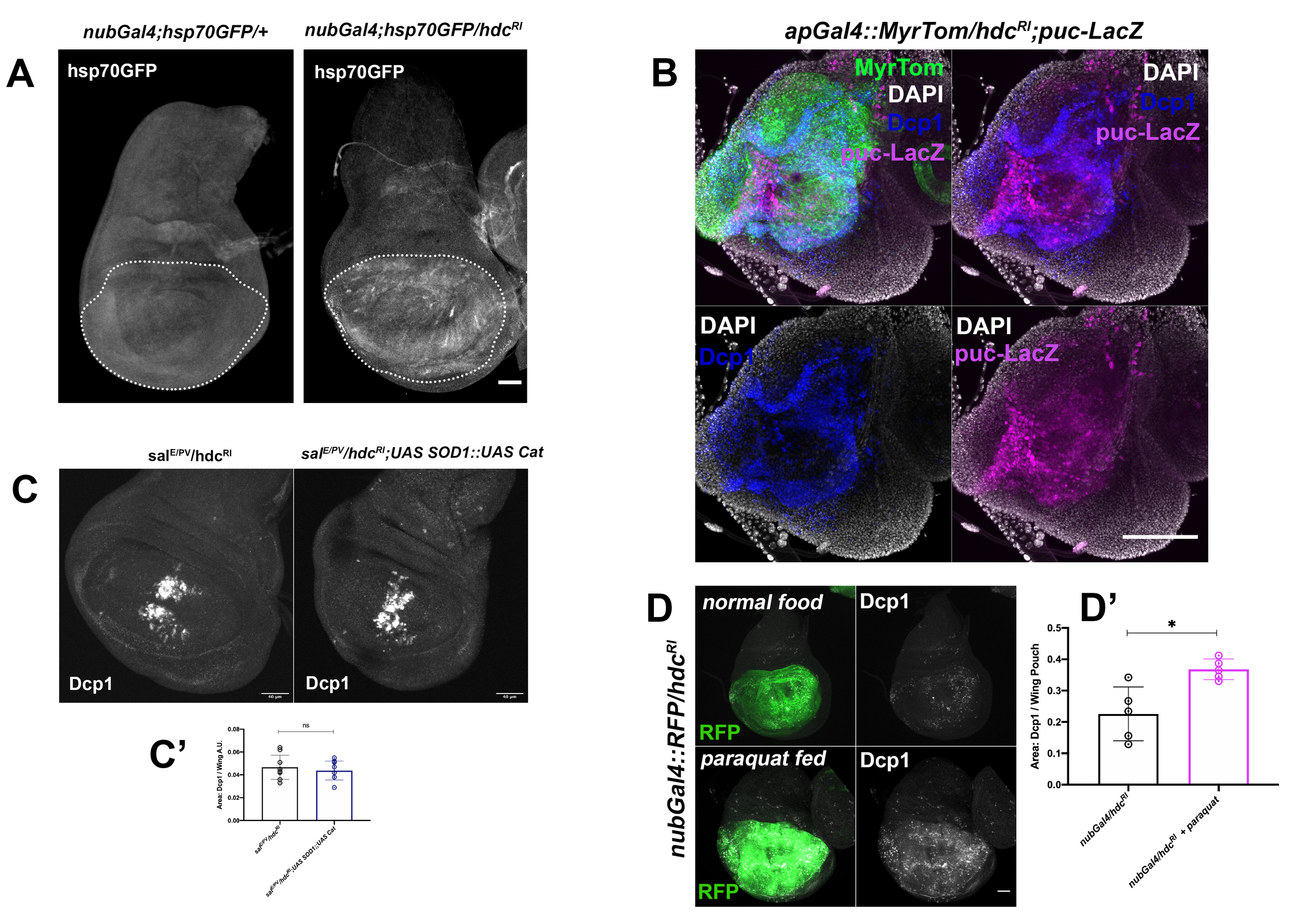

Supplement: S3 Fig — (A) Cells depleted for hdc activate heat shock chaperones. Hsp70 promoter shows enhanced activity in the pouch region of the wing disc of nubGal;hsp70GFP/hdcRI compared to the nubGal;hsp70GFP/+ control, used here to monitor the endogenous activity of the promoter in wild type conditions. (B) The JNK activity marker puckered (puc) is shown to be activated in the apterous region of apGal4::MyrTom/hdcRI L3 wing discs where hdc is knocked down. Puc-LacZ used to mark puc expression (magenda) and Dcp1 (blue) to mark apoptotic cells in the apGal4 region. Scale bar, 40 μm (C, C’) Enzymatic depletion of O2—and H2O2 by overexpression of Catalase and SOD1, together with hdc RNAi, fail to rescue the apoptosis induced by the absence of hdc. Comparisons of the ratios for the Dcp1-positive area / total wing disc area between salE/PV/hdcRI and salE/PV/hdcRI; UAS SOD1::UAS Cat wing discs showed no statistically significant differences in (C’). Unpaired t-test, Welch’s correction (p = 0.51). n = 10 / group. Error bars indicate SD of means. Scale bars, 40 μm. (D,D’) hdc acts protectively against tissue damage in conditions of cell stress. Comparisons of the ratios for the Dcp1-positive area / wing disc pouch area between wing discs from nubGal4/hdcRI L3 larvae fed normal food and nubGal4/hdcRI L3 larvae fed the oxidative stress agent paraquat for 12 h show higher levels of apoptosis in the wing pouch area of the latter (D’). Unpaired t-test, Welch’s correction (*p<0.05). n = 5 / group. Error bars indicate SD of means. Scale bars, 40 μm. (TIF) [file pgen.1009362.s003.tif]
