# Supplementary material for: Systemic and local effect of the Drosophila headcase gene and its role in stress protection of Adult Progenitor Cells
Source: PLoS Genet. 2021 Feb 8;17(2):e1009362. doi: 10.1371/journal.pgen.1009362 (PMC7895379; doi:10.1371/journal.pgen.1009362)
Supplement: S1 Table — (DOCX) [file pgen.1009362.s004.docx]

| Primer | Sequence (5’ – 3’) |
| --- | --- |
| hdc Forward | CTCTCATCGCTGGCCCAATC |
| hdc Reverse | GTGCGTCCCTCGTATTTAACCT |
| Hr3 Forward | GAGGCTTTTCAATCTGAGCATGAA |
| Hr3 Reverse | CGATTCCATGTGCAAGATGGAAAT |
| Hr4 Forward | CGTTTGCATGATCTGCGAGGACA |
| Hr4 Reverse | TTTGGTTATCTCGCAGGTGCCGT |
| Br-C Forward | CATCTGGCTCAGATACAGAACCT |
| Br-C Reverse | CTTCAGCAGCTGGTTGTTGATGT |
| Actin Forward | GCGTCGGTCAATTCAATCTT |
| Actin Reverse | AAGCTGCAACCTCTTCGTCA |
| Tubulin Forward | TGTCGCGTGTGAAACACTTC |
| Tubulin Reverse | AGCAGGCGTTTCCAATCTG |
